# Personal Dedications to David P. Farrington

**DOI:** 10.1002/cbm.2384

**Published:** 2025-04-03

**Authors:** Maria Ttofi, Adrian Grounds, Keri Ka‐Yee Wong

**Affiliations:** ^1^ Institute of Criminology, Faculty of Law University of Cambridge Cambridge UK; ^2^ Department of Psychology and Human Development Ringgold Standard Institution University College London London UK

David Farrington's death means a great loss to us personally, to the academic community and to the people he sought to understand. Donald West introduced him and their longitudinal study of Camberwell boys to one of us in the 1960s at the Institute of Criminology, University of Cambridge. The study became *The Cambridge Study in Delinquent Development.*

David's passion for the scientific study of the causes of crime already shone through and was very impressive. He never wavered in his enthusiasm nor in his dedication to that cohort study, despite branching out into more experimental work, including randomised controlled trials of interventions to ameliorate some of the problems so clearly recognised. The Camberwell/Cambridge study is among the most impressive in the world for its completeness at each follow‐up stage and that was due to the care with which David forged a bond with the boys as they grew into men so that they felt like true participants in a journey of discovery. They then introduced him to their sons and grandsons such that the study became remarkable also for its intergenerational perspectives. It has produced a large number of papers and books—which should be a politician's guide to reducing the burden of crime but which has proved very difficult to get centre stage in spite of his many efforts to do this.

Through this work, David also linked to other longitudinal studies worldwide. We were always immensely grateful that, as a result, we had David to provide the foundation chapter in our textbook of forensic psychiatry for the main sections on understanding pathways into criminal behaviour. Only through such prospective study can we truly understand whether and how the many forms of experience and of mental disorder that may contribute to criminal behaviours actually do so.

No dry academic, David was always a good friend and a cheerful role model. Lunches in the delightful village of Granchester were both memorable and educational. He frequently warned against allowing bureaucracy to interfere with research, and he was proud of the fact that he never undertook any administration that might divert him from his research. How we still need his clear thinking on this. Perhaps universities would struggle less with their finances if this call were heard more clearly. Perhaps fewer researchers would find their grants at risk if ethics approval had not become such a tortuous process, extending way beyond independent ethics board consideration if any health component is included.

In the 1970s, we started planning a journal that would bring together the scientific aspects of forensic psychiatry, criminology and forensic psychology. With David, one of us (J.G.) approached several publishers. Only Wiley was at all interested but they insisted on production first of two or three yearbooks—to prove that there was a market for such, to them, abstruse activities. This we did in 1995, one on *Aggression and Dangerousness* and another on *Reactions to Crime: The Public, the Police, Courts and Prisons*. Even then, Wiley turned down the idea of a journal. Fortunately, the three of us then found Colin Whurr, who was just starting his own publishing company and was delighted by the journal proposal. David was insistent on the journal title: *Criminal Behaviour and Mental Health.* It began with the tripartite editorship, and David never lagged in his enthusiasm for it; his particular skill was in commissioning and delivering first‐class special issues. Ironically, Wiley then took over the journal when Colin retired. Despite his motor neurone disease, David continued to work until the end, while also ensuring that we could welcome Maria Ttofi in his stead to join us and Mary McMurran as the core editorial team together with a wonderful board. It is fitting that these themed issues are a tribute to him—and it is truly wonderful that although we had tentatively proposed one issue, the response was so great that we have filled two. David, we will not forget you nor your work.

***Pamela Taylor and John Gunn***

***John Gunn, Emeritus Professor of Forensic Psychiatry, Institute of Psychiatry, King's College, London, UK***

***Chair, Crime in Mind***

***Pamela J. Taylor, Professor of Forensic Psychiatry, School of Medicine, Cardiff University, UK***

David Farrington had a fantastic career. He started with a PhD in Psychology and became interested in the rich variety of issues involved in the many problems surrounding crime and the criminal justice system.

This led to a phenomenal scope of activity involving teaching an astonishing array of students, a set of publications that generated 148,600 citations when last measured and will certainly grow considerably into the future. In recognition of his expertise, he has served on and led a wide array of policy commissions in all aspects of crime and delinquency, mental problems, justice and incarceration. This recognition was, of course, mostly in the United Kingdom, but almost to the same degree in the United States. Indeed, he was a consultant even to the Pittsburgh Project with Rolf Loeber, and I would host David in my Pittsburgh home, and our discussions would extend well into the night. He and I have spent much time together, and we brought our respective disciplines, his in psychology and mine more remote in operations research, but we were both deeply immersed in the many problems of criminology. He was clearly the champion in those discussions, and I learnt so much from him. David Farrington was an astonishing achiever in all aspects of criminology, and his departure will be profoundly missed—by me and very many others.

***Alfred Blumstein, J. Erik Jonsson University Professor of Urban Systems and Operations Research, Emeritus, Carnegie Mellon University***

Dear David—here I am, trying to find the right words to express all my feelings… is that even possible, I wonder?! Well, reflecting on our ‘standard discussions’ across years, I guess I should start by saying, ‘How are you, boss?!’ You would always giggle when I said that. David, no words could adequately express all the things I want to say. David, thank you!

Thank you for being a fantastic mentor, thank you for all your support, thank you for your kindness, thank you for your patience, thank you for all your knowledge, thank you for all the time you have given me and thank you for our agreements and disagreements. I am grateful that you have taught me how to stand up for what is right no matter what (I try to do this with my own students) and for your relaxed and positive attitude towards life. Your positive energy was contagious. I still remember the joyous dinner on a roof terrace in a restaurant in Cyprus and fantastic ‘down‐to‐earth’ discussions with you and Friedrich over three bottles of red wine! I am grateful for your fantastic sense of humour: ‘Now, Maria, the good thing about Washington DC is that the Greek architecture here is as good as that in your home, but monuments here do not fall apart’!

When you fell ill, I had to accept that I could no longer walk down the corridor from my office to yours. However, travelling to your home address was a good compromise; it made me feel at home, just like you and Sally always made me feel at home in those last few months! And then, 5th of November… You always challenged me on issues of faith: ‘How can you believe in something that cannot be proven? What is the evidence?’ at which point I would always argue that my belief in the afterlife was not ‘irrational’ but rather ‘over‐rational’: different theoretical frameworks.

Well, guess what, David? After your passing, my conviction about the afterlife is even stronger. I refuse to be in a headspace where you don't exist. And for those nonbelievers, all I can say is that ‘your legacy will always stay with us’. David, you rocked the scientific world of criminology like a true rebel… and (I suspect) chose to leave us on the 5th of November, like a true rebel! This is fantastic!

Every year, on Midsummer Common grounds here in Cambridge, I will be looking at the lit‐up sky, enjoying the fireworks, knowing that you are looking at us from above. ‘The world is your oyster, Maria’, you always told me (and all my fellow students). David, my dearest mentor: ‘The sky is your oyster’. Goodbye, for now, David.

***Maria Ttofi, University Associate Professor of Psychological Criminology, Institute of Criminology, University of Cambridge***

***CBMH, Guest editor***

When I arrived at the Institute of Criminology in 1987 as a newly appointed (and anxious) university lecturer, David Farrington was immensely welcoming and encouraging. The structure of the Victorian house with the red door on West Road that was the Institute's home at that time lacked the social spaces that facilitate informal contacts, and David's office was up on the very top floor, but whenever we met, there was always his cheerfulness and laughter. With typical generosity, he was pleased to help me in putting together a course on ‘Psychiatry and Crime’ alongside his on ‘Psychology and Crime’; and throughout his extraordinarily illustrious career, he always remained a great friend to the small academic forensic psychiatry community. His support and encouragement of his research students were remarkable and has resulted in a huge and international legacy of scholarship. Five years ago, he gave a talk to the PhD student group on ‘My career in criminology’. The room was packed. David recalled his early steps in research, not having a career plan, but taking a post in 1969 to assist Donald West with data analysis in the Cambridge Study in Delinquent Development. He could not foresee what would follow. Decades later, his indefatigable enthusiasm and his messages to younger colleagues about taking chance opportunities, learning from mentors, prioritising research and not giving up will be remembered as reassuring, inspiring and of abiding value.

***Dr Adrian Grounds, DM FRCPsych, Honorary Research Fellow, Institute of Criminology, University of Cambridge***

***CBMH Guest editor***

Dear David, I was never your student, yet you have *always* been a great mentor to me because I was Adrian's student. You are one of a kind. Always agreeing to help ECRs and others—and this is reflected in your monthly emails, which capture your wide‐ranging impact and extensive collaborations with students and colleagues worldwide. Your monthly emails always inspire me (and Home Office colleagues, I am told!) to get back to my papers and stick with the evidence! You proposed me as a journal editor and always gave sound advice. You even agreed to write a supporting letter for my promotions despite feeling poorly from your eye surgery, and you still insisted that we have a quick Zoom call so you could understand how best to help me. I was successfully promoted because of you. I will always remember our dinners with Maria/Adrian at Loch Fyne; celebrating your birthday; the energy and enthusiasm with which you would deliver papers at ASC, often back to back, and always carefully prepared with printed notes. You've shown me what kind of academic I would like to be: humble, always listening, kind, humourous and with gravitas. And for this, I am forever grateful, and I hope I can be even half the great mentor, academic and human that you are.

***Keri Wong, Associate Professor of Developmental Psychology, University College, London***

***CBMH Guest editor***

There is no word that can accurately define the kindness and dedication that Prof. Farrington has always shown as a person and a professional. His legacy has been a clear example of how wisdom, perseverance, altruism and effort are combined with the only purpose of teaching and transmitting knowledge with the humility that has always characterised him. Personally, I consider myself very fortunate to have been able to meet such an eminent person as Prof. Farrington, who welcomed me during my first research stay at the University of Cambridge and extended his hand to me. The physical distance between us had not prevented us from keeping in touch over the years, attending international events and publishing together. There are people in life who shine on their own; David was one of them. He has left a gap in our lives that is not easy to fill. The experience, affection or unconditional support that he has given to us is huge. Prof. Farrington has been a gift in our lives, we feel ourselves fortunate to have known him. THANK YOU DAVID!

***Dr Marta M. Aguilar‐Cárceles***

***Associate Professor at the International University of La Rioja (UNIR), Spain***

***Forensic Psychologist in the Justice Administration, Spain***

What impressed me most about David, back then when I first met him in the late 90s and still today, was his unwavering dedication to the *Cambridge Study in Delinquent Development* and its participants. His respect for each and every man who took part in its regular assessments, coupled with his diligent use of the data to generate new knowledge, has truly stood out. I will miss his regular emails listing all his publications in the past month. David's pioneering work on bullying and longitudinal research has left an indelible mark on the field of criminology and far beyond. His compassion and integrity as a human being will continue to inspire those who, like me, will continue to follow in his footsteps.

***Professor Louise Arseneault, Professor of Developmental Psychology, Kings College, University of London***

*To be celebrated as one of the greatest scientists of our time while also being remembered for his remarkable personal qualities is a truly rare and inspiring legacy*. David was far more than an exceptional mentor to me; he was a guiding presence not only in my career but also in shaping the person I have become. He made me a better scholar and, more importantly, a better mentor. As a role model, he taught me that one of the greatest achievements of a researcher is the ability to inspire others—not only through excellence but, above all, through genuine care and unwavering support. He showed me that truly investing in students and their projects, with sincerity and generosity, is one of the most meaningful contributions we can make. He was the best person I could have encountered during my doctoral journey, leaving an indelible mark on my life and shaping the way I am today. His kindness, wisdom and warmth touched countless lives, and I feel profoundly privileged to have known him.

***Dr Miguel Basto Pereira, University Lecturer, William James Center for Research, Ispa – Instituto Universitário***

Since David passed in November 2024, I often find myself still expecting an email from him suggesting a novel idea to investigate. His insight and wisdom were so unique, and being lucky enough to know David and being able to learn from him was truly exciting. David helped me navigate the intersection between criminology and psychology and facilitated me in finding my own path. There are so many important lessons that David has taught me through the years, both as a scholar and as a person. One that stands out is the importance of surrounding yourself with good people, and David was the very best. Where words fall short, I turn to ABBA. Dear David: ‘*So I say thank you for the music, the songs I'm singing, Thanks for all the joy they're bringing, Who can live without it? I ask in all honesty, What would life be?, Without a song or a dance, what are we?, So I say thank you for the music, for giving it to me*’—‘Thank you for the music’ by ABBA (Benny Andersson and Björn Ulvaeus).

***Dr Henriette Bergstrøm, Senior Lecturer in Forensic Psychology, University of Derby, UK***

David's wisdom and guidance left a lasting mark on my life. As an undergraduate, my criminology books were full of his work. Meeting him in real life—and later becoming his PhD student—was beyond exciting. David was not only an outstanding academic mentor but also a generous, wise guide in life. Two of his lessons have stayed with me and continue to shape my life beyond research. Lesson 1 was on focusing on what truly matters. David often said, ‘*If you want to do research, don't waste your time on administrative tasks—you'll never have time to do research!*’ From this, I learnt the importance of focus—prioritising what truly matters and guarding my time from distractions. Lesson 2 was on how perseverance pays off (even if the path is not always straightforward). He also said, ‘*Your article was rejected? Don't worry; just submit it to another journal. Eventually, it will get published*’. I was amazed that such a celebrated scholar had also faced rejections and simply resubmitted his work. It made him wonderfully down to earth. And of course, I will never forget dancing for hours at conferences and how David took us (poor) students out for lunch. David, I miss you and I am forever grateful that our paths crossed.

***Dr Sytske Besemer, UX Researcher and Employee Experience Lead, Cradle***

It is difficult to come to terms with the sad news about the passing of David P. Farrington. David was my academic mentor. He supervised my MPhil/PhD research at the Institute of Criminology, University of Cambridge. I first encountered David's work in 2001 in Ghana when I decided to embark on my postgraduate studies. His name would consistently appear when I searched for people I could work with in criminology. As a student at Cambridge, I was keenly interested in David's lectures. I was fascinated by his intellect and clarity of thought. His depth of knowledge and insight on the development of offending was unparalleled. He was always kind, gentle and generous with his time as a supervisor and mentor. He reminded me that I was his first PhD student from Africa and that meant a lot to me. He encouraged and guided me through my postgraduate studies and career in criminology, starting as a Junior Research Fellow at Clare Hall, Cambridge, to my current post. It has been an honour to know him and to be guided by such a great mind and a wonderful person. His passing is a great loss to criminology and psychology, and especially to his family, students, colleagues and friends. Rest well, David.

***Kofi E. Boakye, PhD, Associate Professor, University of Leicester, Visiting Researcher, Institute of Criminology, University of Cambridge***

All criminologists know that David Farrington was a research scholar of the highest international calibre, especially in the fields of life‐course criminology and of crime prevention. What has received less comment is that David's entire academic career was spent in a single academic department—the Institute of Criminology at the University of Cambridge. The Institute was created only in 1960, so it was still establishing itself, with only a small staff, when David joined it in 1969 as a postdoctoral researcher on the Cambridge Study in Delinquent Development (CSDD). From 1981, David took over from Donald West as the Principal Investigator of the CSDD, and in 1992, the University of Cambridge awarded him a personal professorship in psychological criminology. He never wanted to be the Institute's Director because he preferred to spend his time on research rather than administration, but his stellar research achievements and his many international contacts brought the Institute huge benefits. He also played his full part in teaching, and he cared a great deal about nurturing the next generation of criminologists, as the many warm tributes from his former students eloquently attest. The strengths of today's Institute are derived in a very significant part from David's massive contributions to it.

***Sir Anthony Bottoms, Emeritus Wolfson Professor of Criminology, Institute of Criminology, University of Cambridge***

In David's foreword, ‘Looking Back and Forward’ in *The Future of Criminology*, a celebration of his life's work and the trajectories of inquiry that it advanced, he paid a tribute to the scholars who influenced him. His two earliest influences—his PhD supervisor and the first director of the Cambridge Study, Donald West—taught him to think and write clearly and to be vigilant of the research process and findings. Upon reading this, I felt grateful that my early and major scholarly influence was David. I learnt a lot from him editorially and intellectually. However, I also learnt the importance of being kind. People remember how you treat them, and the many heartfelt tributes and dedications to him since his passing are a testament to that. He set the standard for how to be as a scholar, and I will always be grateful.

***Laura Bui, Senior Lecturer, University of Manchester***

David, I hope you know how much you have always meant to me. Throughout my career, you were always there to support me. In graduate school, when I questioned my abilities, you dispelled my fears. When I worried about career moves, you advised and encouraged me. Whenever I doubted myself, you reassured me. Spending time with you was my favourite part of ASC, whether we worked on research or combed through the book room together. I loved watching your face light up with pleasure while we discussed our latest project and enjoyed your laughter when I joked that you were a ‘Luddite’ because you refused to edit anything on the computer. Hearing you say ‘You do good work’ or ‘I'm proud of you’ was the best reward I ever earned. I have learnt so much from you, not just about criminology but about how to be a good colleague and person. You influence me whenever I work with my own graduate students, trying to apply your lessons and be a good mentor to them in turn. Thank you so much for all your time and energy and for helping to make me the academic and the person I am today.

***Ellen G. Cohn, Associate Professor, Department of Criminology & Criminal Justice, Florida International University***

David Farrington was, for me, an encounter with good fortune that few experience in their academic careers. Chance seating next to David at a meeting of violence and mental health academics, with a both unlikely and unprecedented availability of funding to compete for, started off a series of wonderful collaborations. This resulted in David shifting the Cambridge study to St Bartholomew's Hospital, London, where it was hosted for a decade and culminated in a joint 1.5 million study of personality disorder, risk assessment and reoffending among prisoners. None of this could have been achieved without David's wisdom and guidance to me personally—a new professor with unbounded ambition but little clear direction until talking it through with David. He was a kind man and generous with his time when you needed it. He is much missed by myself and members of my team from that long and fruitful period of our research.

***Jeremy Coid, MD, FRCPsych***

***Emeritus Professor of Forensic Psychiatry, Queen Mary University of London, UK***

***Formerly Professor of Psychiatric Epidemiology, Sichuan University, China***

Although his legacy as a renowned criminologist is indisputable, his other, possibly less well‐known, legacy was as an incredible mentor. David's generosity and kindness were unparalleled. He inspired courage in the face of uncertainty, both vigilance and creativity in research, and faith that he would speak the truth even if it was uncomfortable. Graduate school and the early stages of an academic career can be incredibly challenging, but his guidance and support made the journey far more navigable. It is my hope that I can carry on that legacy in my work with my own doctoral students. He is deeply missed.

***Barbara Cooke, PhD, Keiser University Graduate School***

David Farrington had such a significant influence in the field of life‐course and developmental criminology that it is hard to imagine the field today without his myriad contributions. In teaching my undergraduate course in life‐course and developmental criminology, which provides an overview of the field, what has been striking is the significance of his early contributions to the importance of longitudinal studies in the early years and then the many areas in which David contributed as a scholar as the field has blossomed. Another lasting contribution is how he brought other scholars together over the course of his long career.

***Ben Edwards, Professor of Child and Youth Development and Longitudinal Studies, Australian National University***

David Farrington has made extraordinary contributions to criminology through his knowledge, wisdom, energy and intellectual curiosity combined with methodological rigour. His exceptional academic work will have a lasting influence on the field, and he has inspired many scholars across the world. Among others, he contributed to the shaping of the Zurich Project on Social Development from Childhood to Adulthood. It is one of the many academic adventures that would never have succeeded without his generous thoughts and ideas.

***Manuel Eisner, Wolfson Professor of Criminology, Institute of Criminology, University of Cambridge***

Dearest David, it is still inconceivable that you are no longer with us, and although I am honoured to write this dedication, it has not been an easy task. There are so many fond memories that I will forever hold dear: from dancing to ABBA in hotel bars around Paris (and you making me sing when we were told off for playing music loud!) to the basement of the Institute when you were the first on, and last off, the dancefloor. You were the most humble, unassuming and kind academic I've ever met and had a way to calm the nerves of everyone you encountered, even though we were all probably a bit starstruck that first time! I'll miss your wisdom and guidance the most, but also your sense of fun, humour and the motivation and inspiration you instilled. Your legacy will live on, David; the world of criminology lost a giant the day you left us. We will remember you fondly in memories made, laughs shared and papers published and not sadly in time lost. Thank you, David—for everything.

***Dr Hannah Gaffney, University of Greenwich***

I never had the opportunity to study under or work directly with David, but when I was a new graduate student working with Adrian Raine, I heard so many positive things about him. Eventually, I met David at the ASC meeting and discovered that not only was he an incredibly knowledgeable and prolific researcher with a great vision, but he was also a kind and generous mentor, full of warmth and humour. Since then, every time I saw him interact with people at ASC meetings, he was either smiling or laughing. A few months ago, I received his usual email update on his latest publications, and in it, David shared that he had been diagnosed with motor neuron disease. I was deeply shocked. Dear David, I will always remember your smile.

***Yu Gao, PhD, Professor of Psychology, City University of New York***

David was the most generous and brilliant mentor one could hope for, and I feel incredibly fortunate to have worked closely with him over the years. His passion and unwavering work ethic set a standard I strive to uphold. He was tireless in opening doors, introducing me to fascinating people over lunch and inspiring me at every turn. David shaped my career and pushed me to achieve more than I ever imagined. However, beyond his brilliance, he was a deeply kind and joyful person. I will always treasure our long conversations over drinks, dancing all night to ABBA and even the time we were asked to leave a hotel in Paris for playing music too loud. He was thoughtful enough to send me a congratulatory video for my wedding—something I cherish deeply. I dearly miss his advice and the comfort of knowing he was just a phone call away whenever I needed guidance. There will never be another David Farrington; he remains the role model I aspire to follow. He was a giant, and we, his students, see further by standing on his shoulders.

***Dr Hugo S. Gomes, Institute of Public Health, University of Porto***

Lee Robins once said, ‘David Farrington is the only person who can write faster than I can read’. He published more research than anyone I have ever known. He was also my mentor and friend. He enriched my personal and professional life throughout my career. We first published together in 1991 and last published together in 2021, thirty years later. He invited me to Cambridge for two sabbaticals and found homes there for my family and me both times. From the beginning he encouraged me to focus on doing science rather than become an academic administrator, a decision I never regretted. He encouraged the development and completion of two longitudinal studies Richard Catalano and I conducted that included nested tests of preventive interventions: the Seattle Social Development Project and Communities That Care. When my commitment to completion wavered, he participated in the publication of comprehensive summaries of both those studies (Catalano et al. 2021; Fagan et al. 2019.) What I appreciate most about David is that he always believed I was among the best. Long after I recognised that I would not be chosen, David continued to nominate me for the Stockholm Prize in Criminology year after year until his death. Who could ask for a better friend?

***Emeritus Professor J. David Hawkins, School of Social Work, University of Washington***

David, you changed my life—for the better!—with a reprint that you sent me by snail mail back in 1996. The article reviewed what was known then about the efficacy of early crime prevention initiatives such as the Perry Preschool Project. That paper got me to musing about what my country of Australia was doing about early or developmental crime prevention. A quick check confirmed that we were not doing much at all, and apart from Triple P, there were no scientifically respectable evaluations at all. I pulled together a team of eminent colleagues from a range of disciplines, which led to a seminal federal government report in 1999, *Pathways to Prevention: Developmental and Early Intervention Approaches to Crime in Australia*. This led in turn to a pioneering project in a disadvantaged community in Brisbane called—not surprisingly—Pathways to Prevention! The wonderful news, which you never heard because you were too ill, is that after 20 years, this project led to a decline of 56% in the onset of court‐adjudicated youth crime in the target community. The paper you sent me has led to vastly better lives for many vulnerable children and families and a new focus for crime policy. Thank you!

***Emeritus Professor Ross Homel, AO, Foundation Professor of Criminology & Criminal Justice, Griffith University, Australia***

David has been a starring figure in my life since responding to my 1997 fax from Hamilton, Ontario, Canada, enquiring about the possibility of doing an MPhil at the University of Cambridge. Somehow, he found the time to encourage me (for 27 years!) and so many other people to apply, to pursue, to analyse, to submit and to disregard ‘ignorant’ reviewers. It is hard to figure out whether I should be most indebted to David for the knowledge he imparted, the practical support he offered, the unwavering passion and infectious encouragement he gave so freely, or the warm introductions to so many good people who continue to enrich my life. All who worked with David will continue to push forward (or back) the boundaries of knowledge in his honour, but without David as co‐author, our ideas might be a bit less well developed and our paragraphs and sentences might be a bit less well structured. David, thank you for being the superstar academic who somehow always found the time to support people—like sending a fax to Hamilton, Ontario, Canada.

***Darrick Jolliffe, Professor of Criminology, Royal Holloway University of London***

As a teacher in the late 1970s, David was inspirational, and as a PhD supervisor, he led by example: committed to scholarship, always humble, ready to guide and to support in a positive, constructive way. Later on, he was a fantastic mentor to share teaching and publications in his office or supervisions over a beer at a pub in Granchester. David did not hand over fish on a plate but taught me how best to fish. Above all, it has been a challenge and an honour to try to emulate some of his achievements. Prevention and intervention, he taught me, start with the individual criminologist being a polymath and drawing on all criminal sciences: criminology, forensic psychology, criminal justice and penology. David best epitomised such a polymath, and the world was his oyster, his impact evident in North America, Australia and continental Europe where he pioneered the establishment of EAPL, whereas in Cyprus, he was instrumental in the very successful implementation in schools in Nicosia of *Communities That* *Care*. I always recall David with gratitude and cannot imagine criminology without him.

***Andreas Kapardis, Emeritus Professor of Psychology and Law, Department of Law, University of Cyprus***

Dear David… You have been the kindest, most generous and most brilliant mentor anyone could ever ask for. On my first day at the Institute, you took me out to the pub for lunch. By the time we returned to the Institute a few hours later, we were already in your office, planning our first article. You were never one to waste time! You gave me three crucial pieces of career advice: (1) try not to fall out with anyone; (2) choose your collaborators carefully and (3) avoid administrative work like the plague! I have expressed my gratitude to you many times over the years, but it never seemed enough. I will forever be indebted to you for all your mentorship, your guidance, your advice and your generosity. Thank you for sharing so much wisdom (and data!) with me over the years, for your contagious intellectual curiosity, and for always looking out for my best interests. I am especially thankful to your wife and daughters, who were kind enough to share you with us for so many years. I hope you are somewhere beautiful, doing what you love most.

***Professor Lila Kazemian, John Jay College of Criminal Justice, CUNY***

My time with David was perhaps shorter than that of others, but I am profoundly grateful for the opportunity to have known him. After meeting at the ASC meetings in Philadelphia in 2017, he warmly accepted my request to join the Institute of Criminology at the University of Cambridge as a visiting scholar and co‐author. His words, ‘Come to Cambridge!’ reassured me in the unfamiliar environment. During our 5 months in 2018, our biweekly meetings were filled with his valuable advice on writing and analysis. He often invited me to pubs and restaurants, creating many joyful memories. In the summers of 2019 and 2022, I returned to Cambridge to work with David, yielding five co‐authored papers, one of which won the 2019 Best Paper Award from the Asian Criminological Society. David was delighted with the award. Our final co‐authored paper was based on David's ideas, but unfortunately, he could not review the completed draft. I will submit it soon in honour of his lasting influence. I will miss his warm smile, precise guidance and constant encouragement. As I deeply mourn his passing, his inspiration lives on.

***Professor Emiko Kobayashi, Institute of Liberal Arts and Science, Kanazawa University, Japan***

David Farrington was my PhD supervisor and mentor for more than 20 years. It is hard to imagine a world without him. In the crunch of uncertainty, for many of us, David was a reliable parachute. We could always ask: *What would David say or think about this?* If he did not know the answer (which was rare), he invariably knew where to look for one. He was incredibly supportive. ‘*Don't give up!*’ he would often say when he sensed you needed a push of encouragement. Among his many great qualities, David was a humble scholar who was generous with his wisdom. It didn't matter whether you were an established scientist or a first‐year student—he was always willing to engage with your ideas. He was a giant who never made you feel small. I will remember David for these things, but most of all, as a warm, caring person who inspired people to learn from one another and enjoy the scenery along the way. He took me on the greatest intellectual journey of my life. I will be forever grateful for his kindness and friendship over the years, especially during my time in Cambridge. Thank you, David. I miss you.

***Dr Christopher Koegl, PhD, University of Cambridge***

Dear David, I still can't believe that you have left us. Many memories come back. You are known to the world as a most prominent scholar, but to many of us, you are the kindest man and a friend who warms people around you. I think of the day when we were together in Hangzhou, China, in Chongqing, enjoying your insightful talk in conferences and the exciting exchange of ideas. Working with you has been a major source of learning. From 2009, when I joined the Campbell Collaboration group, we met with other smart minds, learning a lot from you. An important collaboration was ‘the antisocial behaviour of 10‐year‐old boys between Zhuhai, China and Pittsburgh, USA’. We have successfully collected three waves of school children survey data, a rare data collection in China. Your internationally prominent scholarship has gone well into Asia as you have given keynote speeches at the Asian Criminological Society Annual Conference and when you published in the Asian Journal of Criminology. Those happy days of being with you and working with you will last forever in my heart—I miss you! My best wishes to you in heaven, where you must be free from illness and full of joy and happiness! Best, Jianhong

***Professor Jianhong Liu, University of Macau***

As I wrote in my obituary for David for the ESC, he was a ‘giant in criminology and a wonderful man’. His huge number of publications is only one aspect of his outstanding achievement. Similarly important is the breadth of his topics. In addition to his landmark CSDD, there are few criminological topics that David had not addressed. He was always my role model with regard to longitudinal, experimental and not too fragmented research. I met David in the early 1980s at an Advanced Research Center in Germany, where I led a project on resilience. Since then, we were in continuous contact. Together we founded the European Association of Psychology and Law in 1990 and became its first presidents. Although David was much involved in America, he also strongly supported many European colleagues. Our scientific and private contact increased when I became Director of the Institute of Criminology at the University of Cambridge in 2005. Together, we published a number of papers, but beyond research, I admired his personality. He was always empathic, supportive, open‐minded, and dynamic. Although he was a stellar cosmopolitan in science, he preserved English common sense and remained grounded in daily life within his family and the Cambridge Institute.

***Friedrich Lösel, Dr. phil., Dr. phil. habil, Dr. sc. h.c*.**

***Prof Emeritus and former director, Institute of Criminology, University of Cambridge***

***Senior Professor and former director, Institute of Psychology, Friedrich‐Alexander‐University Erlangen‐Nuremberg***

There can be few people within the world of criminology who have not read an article, chapter, book or review written by Professor David Farrington. One of the most prolific authors of our generation, David published widely on topics including offending and victimisation, human development and criminal careers, risk and protective factors, intergenerational criminality, and intervention and prevention, to name but a few. His research had widespread appeal as it was based on the most robust empirical data and spanned temporal, geographical, disciplinary and theoretical boundaries. His regular email correspondence was peppered with delights, promising ‘recent papers that might interest you’, ‘older papers that might be of interest’ and even ‘forthcoming publications to look out for’. His propensity for writing (which he, surely, had a theory to explain) spoke not only of his own intellectual prowess, but also of his capacity to collaborate and share with others. A plethora of scholars, across the globe, can boast co‐authorship credentials and, no doubt, have interesting and amusing tales to tell! For David was incredibly generous with his time, his ideas and his data. In an academic world that is often fraught with intellectual jealousies and competitive rivalries, David sat above such petty matters and was happy to forge a collective path to theoretical enlightenment. However, we must not only remember him as a serious scholar. He was also a fine dancer, as many who attended the earlier European Society of Criminology conferences could attest. The conference dinner in Liege in 2010 springs to mind as a particular exemplar of his prowess on the dance floor. We shall remember David as a jovial, down‐to‐earth, kind‐hearted spirit who was always willing to answer an email, offer advice or point us in the direction of a notable source (whether or not it was written by him). It may be a long time until we meet his likes again.

***Susan McVie and Lesley McAra***

***Susan McVie, Professor of Quantitative Criminology, University of Edinburgh***

***Lesley McAra, Professor of Penology, University of Edinburgh***

As long as I have been in the field of criminology, that is, since 1981, David Farrington has been in the field too. Since I first met David when I was a starstruck PhD student, he has always been there out ahead of me, guiding the field, showing us what questions are interesting and important to work on, setting the pace and encouraging us. He has always been there as a generous mentor I could go to for career advice and a sunny optimistic friend I could count on to tell me again that criminology research is meaningful and important. This special kind of man is virtually impossible to replace. We are all really going to miss him for a long time.

***Terrie E. Moffitt, MBE, PhD***

***Nannerl O. Keohane, University Professor, Duke University***

***Professor, Institute of Psychiatry, Psychology & Neuroscience, King's College London***

During my PhD (2002–2006) and subsequent collaborations with David, three things truly stood out. First, his infectious scientific empiricism steered the field and inspired dozens of students. There is no question my own career would have been vastly different without experiencing David's unwavering commitment to a ‘scientific’ approach in criminology and his advocacy for robust longitudinal and experimental research. Second, David's personal encouragement of young scholars was both generous and touching, especially given his global academic commitments. I remember marvelling with a fellow student at the detailed feedback he would always give on our work—not just on the scientific ideas but also on every detail down to the last comma! He was never too busy for us. Finally, David was simply a kind person. In addition to his consistent positive support of our work, he always took us students out for lunch every term, and his company at conferences was, of course, a highlight. His positivity, both professionally and personally, was contagious, leaving a lasting impact on many lives. Thank you, David.

***Joseph Murray, Professor, Postgraduate Program in Epidemiology, Federal University of Pelotas Director, Human Development and Violence Research Centre, Federal University of Pelotas***

David was an intellectual giant. I will leave it to others to describe his seminal contributions to developmental criminology, public health, psychopathology, evaluation, and public policy. Instead, I will comment on two of his most conspicuous characteristics as a human being—his generosity and respect for others. David was generous with both his time and one of his most valuable scholarly assets—the data from the Cambridge Study in Delinquent Development (CSDD). Many researchers who have assembled longitudinal datasets treat it as an asset to be sparingly made available to others. Not so David—he freely made data from CSDD available to others. Indeed, my first application of group‐based trajectory modelling was based on CSDD data that David had made available to me with no strings attached. A second notable characteristic was the respect David showed to all regardless of status. Snobbery and arrogance were foreign, indeed incomprehensible, to David Farrington. His was a life well lived. He will be missed but will remain as an enduring model of how scholarship should be conducted and how, more broadly, human beings should conduct themselves.

***Daniel S. Nagin, Teresa and H. John Heinz III University Professor of Public Policy and Statistics, Carnegie Mellon University***

Professor David P. Farrington is regarded as the ‘pioneer of Developmental and Life‐Course Criminology’, paving the way for numerous developmental psychologists and criminologists around the world. He was credited with many breakthroughs within his field, and his career laid the foundations of a novel theoretical approach to criminal careers, postulating that the core issue of developmental criminology is to advance knowledge in **how** and **why** individuals exceed normative levels of offending in terms of the frequency and types of crimes committed, between the expected beginning in childhood and the expected ending during middle adulthood.

In coincidence with the most outstanding scientists over centuries, such as Sigmund Freud, Sheldon and Eleanor Glueck and Michael Rutter, Professor David P. Farrington emphasised that the quality of a person's first years of life is relevant in their later development, even when it comes to the development of a criminal career.

His legacy is so broad and outstanding that he inspires, mentors and teaches students, practitioners and colleagues around the world how to prevent delinquency and later recidivism. Professor David P. Farrington's outstanding contribution to the prevention of delinquency and later recidivism brings hope when it comes to interventions designed to prevent criminal careers.

***Mirian Susana Orlando‐PhD, PSYT, BA, MA, National Supreme Court of Argentina***

***Chair of the Section Youth in Conflict with the Law of the Argentina Association of Mental Health‐Member of the World Federation***

***Member of the Editorial Board of Criminal Behaviour and Mental Health (CBMH)***

***Member of the Division of Developmental and Life‐Course Criminology, American Society of Criminology***

As I sit to write this personal note for David, words cannot convey my humbleness enough that David listed me as one person from whom he would like to receive such words. I was never a ‘Cambridge’ student of David's, but I was his student, colleague and friend from afar. I first met David at an NCOVR meeting, and he gave me the time of day. Subsequently, he would always seek me out at meetings as I traversed my graduate career and started in the academy. Who knew then that we would write several books and multiple articles using data from the Cambridge Study. Publications (and citations) are all fine and dandy, of course, but what remains after the acceptances and rejections was our friendship. I have fond memories of David dancing all night long as I played bass in Larry Sherman’s Hot Spots band at the ASC meetings, but also that he would schedule some time with me at every meeting. What I will take away from David is his laughter, smile and sincerity. I thank his family for sharing him and his time with me and the rest of the world. David—may you enjoy many Everton wins and keep on smiling.

***Alex R. Piquero, PhD, University of Miami***

As an undergraduate, I discovered David's work in my developmental and life‐course criminology course and was inspired to apply to the University of Cambridge for my MPhil. I was his supervisee during my MPhil year in Cambridge. I was amazed to learn that such an esteemed scholar was also an incredible person and mentor. David once shared that he decided which research topics to pursue based on what he found interesting. When faced with competing priorities, this valuable insight has inspired me throughout my career. Although David had countless friends and colleagues at ASC, he never failed to greet me with a warm smile and genuine care and kindness over the years. I will be forever grateful for his mentorship and lasting impact.

***Jill Portnoy Donaghy, PhD, Policy Researcher, RAND***

The first time I ever heard David speak was when he came to the University of York (UK) to give a departmental colloquium on criminal offending in 1978. It was a masterful presentation, and I still remember an incredulous question from one senior lecturer who just could not believe David's report of the rate of self‐reported offending that existed in England at that time. David had that impish habit of questioning your basic assumptions on crime! Many years later, at David's retirement function at the Institute of Criminology at the University of Cambridge, I remember him telling us all that progress was not due to him alone and that we all stand ‘on the shoulder of giants’. I did not believe him—he was being far too modest. David was himself a giant, a huge giant, and today we all stand on his shoulders in our attempts to better understand the causes and cures of crime. David was pivotal in jump‐starting my career, serving as a consultant on my very first NIH grant, which incorporated many of his ideas and suggestions, which he so freely gave. I have always been indebted to him, and I dearly miss his advice and good counsel.

***Professor Adrian Raine, Professor of Criminology, Psychiatry, and Psychology, University of Pennsylvania***

Dear David, I first heard of you in the distant eighties as the then‐recognised Professor Farrington, a leading researcher in legal psychology and criminology. Later, I had the opportunity to meet you and follow you as a great teacher in many seminars and conferences in Nuremberg, Manchester, Pamplona, Barcelona, Washington, San Francisco, Brisbane… Then, when I was with you in Cambridge, without ceasing to be the admired professor and great teacher, you were already a close friend. However, always, in every place and circumstance in which I was fortunate to meet you, your teaching, your tireless motivation, your joviality, your kindness and your affection. Now, as I write these farewell lines, my mind vividly evokes your friendly face that welcomes, your affable voice that suggests, that offers, that jokes, that laughs… Memories of you like these, of so many shared efforts and affections, will continue to live, as in me, in the memory and hearts of your many disciples and friends. Farewell, dear teacher and friend.

***Santiago Redondo, Professor of Psychology and Criminology, University of Barcelona***

David P. Farrington's research shaped our understanding of crime and development across decades and continents. His pioneering Cambridge Study in Delinquent Development, following London children as they grew up, fundamentally transformed our knowledge of how delinquency emerges and evolves over the life course. A scholar of eminence and grace, David set an enduring standard for researchers worldwide. Although his wit, energy and intellect will be deeply missed, his lasting contributions to criminology will continue to guide generations of scholars.

***Robert J. Sampson, Woodford L. and Ann A. Flowers University Professor, Harvard University***

Never in a million years did I imagine that one day I would meet David Farrington, whose work I religiously studied as an undergraduate student. Never did I imagine that I would become his MPhil student and that he would be the one to suggest I apply for a PhD, something I never considered before. He saw potential where I could not and sent countless reference letters to a myriad of studentships, determined to find a way to help me fund my PhD. To me, David was much more than a criminologist. He was more than a mentor. He was a constant source of encouragement and optimism. The amount of trust, patience, curiosity, genuine interest and long‐lasting support he was so generously giving me during (and after) my PhD was extraordinary. It was a true honour to work with him, dance to ABBA with him (and with so many people he gathered around him, many of whom I now call friends), show him around Zagreb and introduce him to Croatian wine that he loved so much. It is a true honour to be writing these words now. Thank you for everything, dear David. I will forever cherish every memory I have of you.

***Ivana Sekol, PhD, University of Sheffield***

I sat at David Farrington's feet when my PhD studies were getting off the ground. As a surgeon, the injury literature was far from sufficient to set the scene for a thesis on the epidemiology of violence. Almost every article I read on the antecedents of intentional injury seemed to have been written by someone called David Farrington. I found his address. I wrote to him. He invited me to the Institute of Criminology. We talked for hours and had lunch. Thus began my career‐changing criminology apprenticeship. He introduced me to his criminology friends and Odds Ratios. He advised that if there was time in my surgery schedule for only one criminology meeting a year, the American Society of Criminology annual meeting was the one. Great advice—I took part every year for 25 years. We collaborated. He shared data from the Cambridge Study in Delinquent Development (CSDD) to allow me to study the relationships between childhood predictors of offending, offending over the life course and illness, injury and other health outcomes. This research broke new ground by discovering links between offending and predictors of offending with registered disability and death by the age of 48. A stream of joint articles followed in the Journal of Public Health, the Journal of the Royal Society of Medicine and Criminal Behaviour and Mental Health, for instance. He warned me of the limitations of qualitative research, subjective commentary on crime masquerading as research and what he saw as distracting committee work. Of an opposite number at another ancient UK university, he told me, quite seriously, that ‘He's never done anything I'd call research’! As time went on, he sent international scholars to Cardiff for their sabbatical. Among others, I met Fred Rivara, professor of paediatrics and head of the Harborview Injury Prevention Research Center at the University of Washington as a result and began a productive collaboration with him too. Our paper describing our discovery that almost half of the youth injured in violence had records of violent offending in the year before and the year after their injury and BMJ editorials followed. David also generously read and commented in some detail on my proposal in the early 1990s that violence should be thought about not just as a criminological problem but also as a public health problem. Published in the Journal of the Royal Society of Medicine, this revealed this new perspective; a perspective which informed, and continues to inform, World Health Organization policy and effective novel public health interventions that prevent the physical and mental health harms caused by violence. David's generosity, selfless support and enthusiasm to make connections between researchers have all been hugely valuable to me and to hundreds of others across the world. His selfless service on international criminology bodies has been exemplary too—to the extent that on the Stockholm Prize jury, for example, he postponed what everyone involved knew must eventually happen, his receipt of the Prize.

***Jonathan Shepherd, Professor of Oral and Maxillofacial Surgery, Security, Crime, and Intelligence Innovation Institute, Cardiff University***

I met David Farrington in 1972, when he was a newly appointed assistant university lecturer at the Institute of Criminology. I had no idea then what a powerful influence he would have on my own research career. Yet of all the academics I met during my diploma course, he was by far the most scientific and strategic thinker. His systematic work on reviewing randomised experiments in criminology inspired me to launch the first experiment in police field arrests, which David subsequently praised and promoted as an example of better science for testing crime solutions. His encouragement on that and so many other projects gave me confidence in the face of many challenges. It is clear to me that without David Farrington, my own life course would have been very different and less useful. His example led me and many others to strengthen our efforts to translate criminological research into criminal justice practices. His influence will cast a long arc over that work for many decades to come.

***Lawrence W. Sherman, KNO PhD DHL PhD, Wolfson Professor of Criminology Emeritus, University of Cambridge, Chief Executive Officer, Benchmark Cambridge***

David was an extraordinary mentor, scholar and researcher whose impact on criminology and beyond is immeasurable. As my PhD supervisor and mentor, he was a constant source of support, wisdom, and inspiration. His encyclopaedic knowledge and insightful guidance shaped not only my work but also the careers of countless students and colleagues. David's prolific research output was legendary. Everyone I spoke to was always in awe of his update emails, which listed an astonishing number of newly published articles, book chapters, and even books—just in the last month! His ability to produce rigorous, high‐quality research at such an incredible pace was unparalleled. Yet, despite his remarkable productivity, he was always generous with his time, offering detailed feedback, encouragement, and mentorship to those fortunate enough to work with him. Beyond his academic achievements, David's kindness and dedication to his students made him truly special. He fostered a supportive and stimulating research environment, pushing us to think critically and aim high. His legacy will endure not only in the vast body of research he leaves behind but also in the many lives he influenced. He will be deeply missed.

***Guy Skinner, PhD, Research Associate, Department of Public Health and Primary Care, University of Cambridge***

It seems a long time ago when I first met David at Cambridge, and he agreed to supervise me. I always found him a thoughtful and compassionate man and with his guidance I was able to achieve my goal. He then encouraged me to continue my research and teaching, and I have always followed his model when supervising my own students. David had a wonderful way of encouraging me to go that extra mile through some difficult personal events, which demonstrated his incredible patience, energy and tenacity. It was indeed a great privilege to work with him.

***Delphine Theobald, PhD, University of Cambridge***

David remained the person I first met in 1976 and worked with numerous times, passionate about what he did, painstaking in how he did it, and simultaneously proud and modest about what he accomplished. He was remarkably fortunate that the passion persisted—in my experience, at least among academics, it seldom does. Many others are fortunate that he shared it with them. We cite‐checked every reference and verified every data point in his first, classic, 1979 *Crime and Justice* article on longitudinal studies. Not a single error—a standard he almost always maintained and that few others, if any, have matched. For nearly 30 years, we were active, if intermittent, partners in crime, doing articles and books together, participating in one another's projects and seeing one another every month or two. He was, or seemed, indefatigable. For decades, he travelled to the United States almost monthly, going from the airport directly into a meeting and, returning home, from Heathrow at dawn to a Cambridge class later that day. I have had the good fortune to work with and learn from many talented people, among whom David was special. Knowing him was a blessing.

***Professor Michael Tonry, External Scientific Member, Max Planck Institute for the Study of Crime, Security, and Law, Freiburg***

When I first met David in 1989, I instantly warmed to him—I was struck by the scientific rigour that he brought not just to his work but to his thinking more broadly and his personal authenticity. I subsequently discovered that we also had quite a lot in common in terms of our backgrounds. Throughout my career in forensic psychology, he has been my mentor—I have joyful memories of working with him on the evaluation of the Military Corrective Training Centre‐based approach to reducing reoffending among young offenders. I remember the rigour and professionalism—but I also have very fond memories of the laughter—and there was plenty of that too. I enjoyed his sense of humour. He was on the appointments panel when I was appointed as Head of Psychological Services in prisons and probation. He subsequently kindly wrote me a reference when I applied to work in the academic sector. And that reflected the giving soul that characterised his approach to working with others, especially with mentoring and supporting new generations of scholars and practitioners. He was such a prolific scholar and leaves a huge legacy for the criminological psychology field and public policy. So missed.

***Professor Graham Towl, Department of Psychology, University of Durham, Formerly Chief Psychologist at the Ministry of Justice***

I first encountered David as a graduate student newly inducted into the field of criminology. Within the poorly suppressed excitement of my matriculating cohort, students marvelled that we were to be taught by leaders of the field *such as David Farrington*. I remember my first seminar on psychology and crime, in which David answered students' questions—including the inevitable and futile effort of a self‐important peer to derail the train of logic we had embarked on—with concise, irrefutably evidence‐laden answers. His ability to empirically answer criminological questions in the classroom and in his vast body of research, his emphasis on the importance of data and a strong scientific approach and his extraordinary productivity made and will continue to make him an inspiration to many aspiring research scholars. It was a privilege to develop as a criminologist—and especially as a developmental life‐course criminologist—within his orbit, our paths crossing from the classroom to conference halls to the pages of journal special issues to meeting rooms in the Home Office to the dance floor at the ASC. His field‐defining work will continue to shape the trajectory of criminology and many criminologists. He will be greatly missed, but his impact on the field will endure.

***Kyle Treiber, University Associate Professor in Neurocriminology, Institute of Criminology, University of Cambridge***

David was the PhD supervisor and mentor everyone wanted. Infectiously enthusiastic. Amazingly patient. Generous with his knowledge, his time, a warm meal, handwritten feedback and a conference dance. Always pragmatic in his advice: ‘Just get it down on paper—suspend your critical judgement—for now’; ‘Don't get bogged down with administrative duties or you'll never have time for your research’. And my personal favourite, said with a twinkle in his eye: ‘Congratulations on your wedding engagement. Just don't change your name—it will scatter your publication record!’ Nearly 2 decades later, if I had a dollar for every time I have said to my own students, ‘my PhD supervisor used to say…’, I would be far wealthier than the average academic! David's formal impacts on the field speak for themselves—the stacks of publications and the lists of awards. I am so honoured to help document some of the more personal impacts he also had. David—you were a force, and you will be dearly missed by all of us fortunate enough to have grown under your wings. P.S. I never did crack the administration/research code. But you'll be happy to know that I'm still working on it…

***Sarah van Mastrigt, Associate Professor, Department of Psychology and Behavioural Sciences, Aarhus University, Denmark***

David Farrington was the most important experimental criminologist of the last half‐century. David was someone who thought out of the box and constantly tried to expand the boundaries of criminology. I asked him once about why he spent so much time in the United States; I believe he noted at one point that he was flying to the United States almost weekly. He told me simply: ‘In the UK when I raise an important new idea for a study, they tell me all of the reasons why it could not be done. But in the US, they would ask what we needed to do to get it done’. I was first introduced to David in the late 1980s by my mentor Al Reiss. I was awed at meeting him, as he was already one of the most important criminologists in the world. He was immediately friendly and supportive, and I looked upon him as a mentor after that. He and I organised a symposium at the Hebrew University that led to a well‐received book on What Works in Crime Prevention and Rehabilitation. We were co‐chairs together of the Campbell Collaboration Crime and Justice Group. David was a pleasure to work with. He was simply a mensch!

***Professor David Weisburd, George Mason University & Hebrew University of Jerusalem***

I was one of David Farrington's PhD students at Cambridge. I matriculated in 1996. It has been said that I spent my formative years at the Institute of Criminology—7 West Road, of course. The criminologist and prevention scientist who I am today is because of David. He taught me what it means to be a scholar. He showed me the ropes. He also made me a better person—a better husband and a better father. He was not direct. Heck, he was a developmental psychologist. He modelled behaviour and shared stories about his wonderful family, all the while giving me a window into how he treated others and how he worked to balance his professional and family life. Jennifer, Ryan, and I would end up going to Cambridge for a sabbatical, to Amsterdam, to other wonderful places—all with thoughts of David and family decamping to Ottawa and Washington in decades past. I offer these few lines in remembrance of David, as a way to capture what he meant to me and what he means to so many of us: ‘Beloved scholar, colleague, and friend. Sorely missed. His brilliance and enthusiasm radiate still’.

***Brandon C. Welsh, Dean's Professor of Criminology, Northeastern University***

David's academic legacy will live on forever. My first contact with David was through a very generous book review he wrote on my PhD. We subsequently came to collaborate on some comparative studies and eventually became colleagues at Cambridge. I will always cherish our pub dinners and illuminating and inspiring discussions of the intricacies of doing longitudinal research. Working with David was like working with a top athlete ‘being in the zone’. His ability to concentrate on the task at hand is, in my experience, unparalleled, and his deep knowledge of developmental criminology was encyclopaedic. I particularly remember when he came to Stockholm to work with me on a comparative study of criminal careers. I thought I would take him to his hotel because it was rather late in the afternoon, but he insisted that we go directly to my office and start working, which we did. I think the best way to capture Davids's great academic standing and celebrity is to share a comment I overheard at an ASC meeting from one young criminologist saying to another young criminologist pointing towards David: ‘That is David Farrington. He is the most famous criminologist in the world’.

***Per‐Olof Wikström, Emeritus Professor of Ecological and Developmental Criminology, Institute of Criminology, University of Cambridge***

*Professor David P. Farrington* passed away on 5 November 2024, leaving an incommensurable void in the life of his family and in those who cared about him deeply. *David*’s intellectual honesty and his sensitivity coupled with his intelligence and knowledge made him not only the «Renaissance Man of Criminology», but also one of the kindest human beings we ever met, I have ever met. If criminological psychology is the sound and interdisciplinary science we know today, it is thanks to *David Farrington*. His prospective longitudinal *Cambridge Study in Delinquent Development*, his endeavour to make criminological psychology a science in the service of humanity, has encouraged many of us to devote our careers to scientific research. What I sense as important to share with everyone is that *David Farrington* was able to unearth the best in everyone who worked with him and that his enthusiasm and commitment to life and research were contagious, so that he will continue to be with us forever. An echo of his positiveness is perfectly worded by William Shakespeare in Act II, Scene 5 of Twelfth Night: «Be not afraid of greatness. Some are born great, some achieve greatness, and some have greatness thrust upon them».

***Georgia Zara, Associate Professor, Department of Law, University of Turin, Italy***

David has been the most amazing mentor in the world. He was a criminology giant with a Nobel‐type prize (the Stockholm Prize in Criminology) and many other prizes. Every single criminologist in the world knows and uses David's work. He had many mentees, but he made each one of us feel special. He always had time for us, remembered exactly what we were working on, never seemed to be in a hurry, and would always take the time to listen. No matter the problem, David always knew the solution, and if we entered his office in distress, we would leave feeling happy and calm. He taught me how to write (he was an amazing writer!), how to design projects and how to bring them to fruition. He always knew the mathematics behind statistical analyses and could solve complex issues with just paper and a pen. He would answer all of our complex, but also many silly questions with patience, making us feel valuable and competent. He would patiently read countless versions of our manuscripts, making corrections in record time. He was always up to date with criminological research, wrote beautiful papers himself, edited several books simultaneously, some special issues, attended conferences (giving amazing lectures), taught, ran data analyses himself… and never missed a single email. David left us way too early, but the impact of his life and legacy is what most people would need dozens of lifetimes to achieve. He must have had some superhuman powers. He was and always will be my superhero!

***Izabela Zych, Professor of Psychology, University of Cordoba, Spain***

